# Deletions of *pfhrp2* and *pfhrp3* genes were uncommon in rapid diagnostic test-negative *Plasmodium falciparum* isolates from Uganda

**DOI:** 10.1186/s12936-020-03547-4

**Published:** 2021-01-02

**Authors:** Sam L. Nsobya, Andrew Walakira, Elizabeth Namirembe, Moses Kiggundu, Joaniter I. Nankabirwa, Emmanuel Ruhamyankaka, Emmanuel Arinaitwe, Melissa D. Conrad, Moses R. Kamya, Grant Dorsey, Philip J. Rosenthal

**Affiliations:** 1 Infectious Diseases Research Collaboration, Kampala, Uganda; 2 Department of Pathology, College of Health Science, Makerere University Kampala, Kampala, Uganda; 3 Department of Medicine, Makerere University College of Health Sciences, Kampala, Uganda; 4 Department of Medicine, University of California, San Francisco, CA USA

**Keywords:** *pfhrp2*, *pfhrp3*, *Plasmodium falciparum*, HRP2, Rapid diagnostic test

## Abstract

**Background:**

Rapid diagnostic tests (RDTs) play a key role in malaria case management. The most widely used RDT identifies *Plasmodium falciparum* based on immunochromatographic recognition of *P. falciparum* histidine-rich protein 2 (PfHRP2). Deletion of the paralogous *pfhrp2* and *pfhrp3* genes leads to false-negative PfHRP2-based RDTs, and has been reported in *P. falciparum* infections from South America and Africa. However, identification of *pfhrp2/pfhrp3* deletions has usually been based only on failure to amplify these genes using PCR, without confirmation based on PfHRP2 protein expression, and understanding of the true prevalence of deletions is incomplete.

**Methods:**

Deletions of *pfhrp2*/*pfhrp3* in blood samples were investigated from cross-sectional surveys in 2012-13 in three regions of varied malaria transmission intensity in Uganda. Samples with positive Giemsa-stained thick blood smears, but negative PfHRP2-based RDTs were evaluated by PCR amplification of conserved subunit ribosomal DNA for *Plasmodium* species, PCR amplification of *pfhrp2* and *pfhrp3* genes to identify deletions, and bead-based immunoassays for expression of PfHRP2.

**Results:**

Of 3516 samples collected in cross-sectional surveys, 1493 (42.5%) had positive blood smears, of which 96 (6.4%) were RDT-negative. Of these 96 RDT-negative samples, *P. falciparum* DNA was identified by PCR in 56 (58%) and only non-falciparum plasmodial DNA in 40 (42%). In all 56 *P. falciparum*-positive samples there was a failure to amplify *pfhrp2* or *pfhrp3*: in 25 (45%) *pfhrp2* was not amplified, in 39 (70%) *pfhrp3* was not amplified, and in 19 (34%) neither gene was amplified. For the 39 *P. falciparum*-positive, RDT-negative samples available for analysis of protein expression, PfHRP2 was not identified by immunoassay in only four samples (10.3%); these four samples all had failure to amplify both *pfhrp2* and *pfhrp3* by PCR. Thus, only four of 96 (4.2%) smear-positive, RDT-negative samples had *P. falciparum* infections with deletion of *pfhrp2* and *pfhrp3* confirmed by failure to amplify the genes by PCR and lack of expression of PfHRP2 demonstrated by immunoassay.

**Conclusion:**

False negative RDTs were uncommon. Deletions in *pfhrp2* and *pfhrp3* explained some of these false negatives, but most false negatives were not due to deletion of the *pfhrp2* and *pfhrp3* genes.

## Background

Malaria is among the leading health threats in Africa. Sub-Saharan Africa carries the largest malaria burden in the world, with an estimated 213 million cases and 381,000 deaths, primarily from *Plasmodium falciparum*, in 2018 [1]. The World Health Organization (WHO) recommends that all cases of suspected malaria should have the diagnosis confirmed by either microscopy or malaria rapid diagnostic test (RDT) before treatment. The WHO gold standard for malaria diagnosis remains microscopic examination of Giemsa-stained thick and thin blood films [1]. This method requires an experienced reader to provide accurate diagnosis. RDTs offer a number of benefits over microscopy, as they are less labour-intensive, do not require electricity, require less sophisticated laboratory personnel, and specifically detect *P. falciparum*.

Over 259 million malaria RDTs have been used in sub-Saharan Africa annually since 2018 [1]. Malaria RDTs can target a number of antigens, including histidine-rich protein 2 (PfHRP2), lactate dehydrogenase, and aldolase [2]. PfHRP2 is abundantly expressed by erythrocytic stages of *P. falciparum*, and it is the antigen most commonly used in malaria RDTs. RDTs that detect lactate dehydrogenase or aldolase have the advantage of also detecting non-falciparum species, but these tests are less sensitive and more susceptible to degradation from heat and humidity than are PfHRP2-based RDTs [3].

Some PfHRP2-based RDTs test positive also in the presence of the paralogous PfHRP3 protein, as the proteins share antigenic epitopes [4, 5]. PfHRP2-based RDTs are more sensitive than those directed against other antigens because of higher levels of circulating PfHRP2 and PfHRP3, superior antigen–antibody binding kinetics, and amplification of secondary antibody binding due to repeated epitopes in the HRPs [6, 7]. PfHRP2-based RDTs have sensitivity and specificity for *P. falciparum* diagnosis similar to those for expert examination of Giemsa-stained thick blood smears [8].

An important limitation of PfHRP2-based RDTs is potential false-negative results due to deletion of the *pfhrp2* and *pfhrp3* genes [9–11]. Existence of *P.* *falciparum* lacking the *pfhrp2/pfhrp3* genes poses a major risk to malaria control programmes because, if these parasites circulate widely, infected individuals may not be diagnosed and treated, and thus may serve as parasite reservoirs enabling continued transmission. Parasites with *pfhrp2/3* deletions were first reported in Peru about 10 years ago, after investigation of microscopy-positive/RDT-negative infections, with subsequent reports from other countries in the America. [5, 12]. More recently, *pfhrp2/3* deletions have been described in *P. falciparum* from parts of Africa, most notably Eritrea, where prevalence is reported to be much higher than in other areas for unknown reasons [13]. Data for *pfhrp2/3* deletion prevalence from other parts of Africa are limited, but deletions have been reported in *P. falciparum* from a number of countries in west (Senegal, Mali) [9, 14], central (Rwanda, Democratic Republic of Congo) [15, 16], and east (Kenya, Tanzania, Uganda) Africa [17, 18]. A previous study of samples collected in Uganda in 2014-15 noted that seven of 116 (6.0%) microscopy-positive/RDT-negative isolates had deletion of *pfhrp2*; two of these also had deletion of *pfhrp3* [18]. Another study of 300 isolates collected from 48 Ugandan districts in 2017-19 reported that 3.3% had deletion of only *pfhrp2*, 3.0% had deletion of only *pfhrp3*, and 3.3% had deletions of both genes [19]. Importantly, in many studies reports of *pfhrp2/3* deletions have been based only on failure to amplify one or both of these genes by PCR, and it is not clear if amplification failure reliably identifies true deletions. Of note, in samples from Peru [12] and Eritrea [13] *pfhrp2/3* deletions identified by PCR were validated by demonstration of lack of expression of PfHRP2 protein [18]. The aim of this study was to determine whether deletions in *pfhrp2/pfhrp3* explain occasional observations of negative PfHRP2-based RDTs in individuals with positive blood smears for malaria parasites. The studied samples were from cross-sectional surveys at three sites in Uganda with positive blood smears but negative malaria RDTs, considering both amplification of *pfhrp2* and expression of PfHRP2.

## Methods

### Source of samples

Cross-sectional surveys, including blood collection, were conducted in 2012 and 2013 in 200 randomly selected households at each of three districts located in different epidemiological settings: Kihihi, Kanungu District, a rural area with relatively low transmission intensity in southwestern Uganda; Walukuba, Jinja District, a peri-urban area with moderate transmission intensity in central Uganda; and Nagongera, Tororo District, a rural area with high transmission intensity in eastern Uganda. Of note, since the time of this study transmission has decreased considerably in Walukuba, likely due its peri-urban characteristics, and in Nagongera, associated with regular rounds of indoor residual spraying of insecticide since 2014 [20]. Participants for this study were children ages 6 months to 15 years who were full-time residents of the recruited households, selected as previously described [21]. This study was approved by the Makerere University Research and Ethics Committee, the Uganda National Council of Science and Technology, and the University of California, San Francisco Committee on Human Research.

### Sample collection and malaria diagnosis

Blood was obtained by finger prick for thick blood smears, malaria RDTs and drying on filter paper for molecular studies. Thick blood smears were stained with 2% Giemsa for 30 min [22] and read by laboratory technologists at the field sites. Parasite densities were calculated by counting the number of asexual parasites per 200 leukocytes (or per 500 leukocytes, if the count was < 10 asexual parasites/200 leukocytes), assuming a leukocyte count of 8000/μL. For quality control, all slides were read by a second microscopist, and discrepancies resolved by a third microscopist at the field sites. In addition, all positive blood smears with parasite densities ≤ 20,000/µL based on the field readings were re-read by an expert microscopist in Kampala; confirmation of parasitaemia was required for inclusion in the final analyses. RDTs (SD BIOLINE Malaria Ag Pf, a PfHRP2-based test from Standard Diagnostics Inc; Suwon City, Republic of Korea) were performed immediately after blood collection following manufacturer’s instructions. Samples for study were all those that were positive for malaria parasites by microscopy but negative by RDT.

### Plasmodium species identification

DNA was extracted using Chelex100, as previously described [23]. Species identification was performed by nested species-specific PCR with primers specific for the 18S small subunit ribosomal DNA gene of all human plasmodial species, as previously described [24]. PCR reactions were performed in 25 µl containing 1 × standard *Taq* buffer (New England Biolabs), 200 µM deoxynucleoside triphosphates, 200 µM of each primer, 2 µl of template DNA (from Chelex extraction or the prior cycle of PCR), and 1 unit of *Taq* polymerase (New England Biolabs). All reactions included negative controls (water) and positive controls, obtained from the Biodefense and Emerging Infections Research Resources Repository (BEI U.S).

PCR products were resolved by electrophoresis on 2% agarose gels stained with ethidium bromide and visualized by UV illumination. Sizes of amplicons were identified based on comparison with standard fragments of known size.

### Detection of deletions in *pfhrp2* and *pfhrp3* genes

To identify deletions, we PCR-amplified fragments spanning exon 1, the intron, and exon 2 of the *pfhrp2* and *pfhrp3* genes, as previously described [5]. *P. falciparum* Dd2 strain DNA was a negative control for *pfhrp2* and a positive control for *pfhrp3. P. falciparum* HB3 strain DNA was a negative control for *pfhrp3* and a positive control for *pfhrp2*. All PCR reactions were performed in duplicate. PCR products were separated and visualized on 2% agarose gels. In the event of discordant replicates, reactions were repeated, and the result recorded was that seen in multiple assays. Deletions were identified by the absence of amplification of *pfhrp2/3* in the setting of successful amplification of ribosomal DNA in the sample and amplification of *pfhrp2/3* in positive control DNA.

### Multiplicity of infection (MOI)

MOI, the number of different parasite genotypes co-existing within a host, is a metric of transmission dynamics [23, 24]. To determine MOI, the *3D7* and *FC27* alleles of the merozoite surface protein-2 (*msp2)* gene, which each have extensive size polymorphism, were amplified as previously described [25]. Amplicons were identified on 2% agarose gels, the size of products was compared to standards on densitometric digitized gel images analysed by GelCompar II software (Applied Maths NV Belgium), and the number of differently sized amplicons in each sample was determined.

### Bead-based immunoassay for detection of PfHRP2 protein

As the absence of gene amplification does not definitively prove the presence of a gene deletion, we also assessed expression of PfHRP2 in study samples. Unfortunately, adequate material was available for this analysis for only 39 of the 56 RDT-negative *P. falciparum* samples. For these assays recombinant PfHRP2 (Microcoat Biotechnologie GmbH, Bernried am Starnberger See, Germany) was used as a positive control and blood from persons not infected with malaria as a negative control. PfHRP2 levels was quantified using a bead-based immunoassay with a MAGPIX instrument (Luminex Corp., Austin, TX), as previously described [26–28]. Briefly, the bead-based HRP2 immunoassay, which relies on antigen capture, is capable of detecting PfHRP2 at sub-picogram levels, allowing fast processing and screening of large numbers of samples. As in prior studies, the cut-off for a positive PfHRP2 antigen result was the mean plus three standard deviations based on a panel of 92 antigen negative blood samples [28].

## Results

### Study samples

Of 3516 samples collected in cross-sectional surveys at three sites in Uganda, 1493 (42%) were positive for malaria parasites by Giemsa-stained thick smear (Fig. 1). Of the 1493 smear-positive samples, 96 (6.4%) were negative by PfHRP2-based RDT. These 96 samples were further investigated.

### Amplification of plasmodial DNA in RDT-negative samples

Of the 96 microscopy-positive/RDT-negative samples, 56 (58%) had *P. falciparum* ribosomal DNA amplified, and in 40 (42%) only non-falciparum plasmodial DNA was amplified. In these samples the species identified was *Plasmodium vivax* in 12 (30%), *Plasmodium ovale* in 10 (25%), and *Plasmodium malariae* in 18 (45%). The range and SD for parasite densities for *P. falciparum* positive/RDT-negative samples were 48 – 3400 (± 660) parasites/µl. The multiplicity of infection for *P. falciparum* positive/RDT-negative samples was low at all three sites (mean 1.5; Table 1).

**Table 1:** Characteristics of study districts and results of *P. falciparum* surveillance

	Kanungu	Jinja	Tororo	Total
Total samples collected	1077	1039	1400	3516
Smear-positive samples (%)	419 (38.9)	189 (18.2)	885 (63.2)	1493 (42.5)
Smear-positive/RDT-negative samples (%)	20 (4.8)	19 (10.1)	57 (6.4)	96 (6.4)
*P. falciparum*-positive/RDT-negative samples (%)	3 (0.7)	9 (4.8)	44 (5.0)	56 (3.8)
Geometric mean parasite density/µL	701	225	289	291
MOI (mean)	1.7	1.4	1.5	1.5

### Amplification of *pfhrp2* and *pfhrp3* in *P. falciparum* positive/RDT-negative samples

To analyse the 56 *P. falciparum* positive/RDT-negative samples for potential deletions in the *pfhrp2* and *pfhrp3* genes, these genes were amplified. For all 56 samples there was a failure to amplify *pfhrp2* or *pfhrp3*. Of these samples, in 25 (45%) *pfhrp2* was not amplified, in 39 (70%) *pfhrp3* was not amplified, and in 19 (34%) neither gene was amplified.

### Immunoassay for PfHRP2 protein expression

Of the 56 *P. falciparum* positive/RDT-negative samples, 39 had adequate remaining material for additional study. These 39 samples were evaluated for expression of PfHRP2 by bead-based immunoassay. With this assay, four of the 39 samples (10.3%) had no detectable PfHRP2 antigen (Table 2). These four samples all had failure to amplify both *pfhrp2* and *pfhrp3* by PCR. Overall, in 35/39 (89.7%) samples with failure to detect *pfhrp2* and/or *pfhrp3* by PCR, PfHRP2 was detected by immunoassay. The mean parasite density for four samples with no PfHRP2 protein expression was 279 parasites/ul, with MOI of 1.8 (Table 2).

**Table 2:** Detection of PfHRP2 antigen by immunoassay in microscopy—positive/RDT-negative isolates

Site	Kanungu			Jinja			Tororo			All districts		
	n (%)	MOI	PD	n (%)	MOI	PD	n (%)	MOI	PD	n	MOI	PD
PfHRP2 not detected	0	0	0	3 (60)	1.0	321	1 (3.0)	1.2	263	4	1.1	291
PfHRP2 detected	0	0	0	2 (40)	2.0	134	33 (97)	1.6	579	35	1.8	279
Total	0	0	0	5 (100)	1.4	246	34 (100)	1.6	570	39	1.7	280

n (%), number of samples; MOI, multiplicity of infection; PD, geometric mean parasite density/µL

## Discussion

To explore the basis of false-negative malaria RDTs, the presence of deletions in *pfhrp2/pfhrp3* genes were investigated in Ugandan blood samples that were positive for malaria parasites by blood smear, but negative by PfHRP2-based RDT. To evaluate for potential deletions in RDT-negative samples amplification of the *pfhrp2* and *pfhrp3* genes, amplification of sub-unit ribosomal DNA (for species identification), amplification of *msp2* (for MOI determination), and expression of PfHRP2 by immunoassay, were assessed following an established protocol [28]. Importantly, no samples characterized as *P. falciparum*-negative by PCR demonstrated expression of *pfhrp2* by immunoassay. In the small subset of samples (6.4%) that were microscopy-positive, but RDT-negative, the false negative RDT results were explained by non-falciparum malaria infection in 42%. The identification of non-falciparum malaria infections was consistent with our recent identification of non-falciparum infections in 8.2% of subjects diagnosed with malaria at 10 sites in Uganda, although most of these were mixed falciparum/non-falciparum infections [29]. For *P. falciparum* samples that were false negative by RDT, only 10.3% had both failure to amplify *pfhrp2/pfhrp3* by PCR and no detection of PfHRP2 by immunoassay, consistent with false negative RDT results caused by absent expression of PfHRP proteins. Thus, the data suggest that in Uganda, *pfhrp2/pfhrp3* gene deletion is present, as described in other recent studies [18, 19], but that absence of PfHRP2 expression was uncommon, and that this absence did not explain most false-negative RDT results.

Deletions of the *pfhrp2/pfhrp3* genes, leading to lack of expression of PfHRP proteins, has been well documented in *P. falciparum* from a number of regions, most notably parts of South America [12] and, in Africa, Eritrea [13]. Lower prevalence of deletions has been reported in *P. falciparum* from many African countries [9, 14–18], including Uganda [18, 19]. However, methods used in these studies have not been consistent. In some studies, only *pfhrp2*, but not *pfhrp3* has been studied, despite the fact that expression of PfHRP3 may yield a positive PfHRP2-based RDT. In many studies, *pfhrp2/pfhrp3* gene deletion has been documented based only on failure to PCR-amplify the genes. However, even with controls demonstrating amplification of other *P. falciparum* genes, there is concern that failure to amplify *pfhrp2/pfhrp3* might be due to technical difficulties rather than true deletions. To address this concern, efficient methods are now available to assess expression of PfHRP2 using a bead based immunoassay [27]. This technology was utilized to further characterize samples with *pfhrp2/pfhrp3* gene deletions based on PCR results [5].

Results from the PfHRP2 immunoassay were revealing. Most samples that had apparent deletions of *pfhrp2* or *pfhrp3* based on PCR actually showed expression of PfHRP2 by immunoassay. This result suggests that failure to amplify *pfhrp2/pfhrp3* was, in some cases, due to technical challenges (with identification of the false negatives facilitated by the high sensitivity of the immunoassay), rather than true deletions. A less likely possibility is that the *pfhrp2*/3 deletions suggested by PCR were real, and that the immunoassay yielded false positive results. PCR might have failed to amplify *pfhrp2/pfhrp3* due to the presence of enzyme inhibitors in samples, PCR primer mismatch due to mutations that did not affect PfHRP2/3 expression, inadequate quantities of DNA for successful amplification, or other technical factors. Consistent with these explanations, our samples were extracted without full purification, potentially allowing some PCR inhibitors in reactions. In addition, parasite densities and MOI were low, and samples were extracted after long-term storage on filter paper, all consistent with limited quantities of DNA for reactions. Results suggest that definitive detection of *pfhrp2/pfhrp3* deletion should incorporate assays for both DNA and protein.

The study had important limitations. First, the study was completed well after collection of samples in 2012-13, and so offers limited insight into the current prevalence of the deletion of *pfhrp2/pfhrp3* may have been identified. Third, this was a cross-sectional study of households, with the large majority of subjects asymptomatic, so it offers relatively little insight into the prevalence of *pfhrp2/3* deletions in those presenting with symptomatic malaria. Fourth, the full *pfhrp2/pfhrp3* genes in study samples was not sequenced, thus the mechanisms behind failure to amplify *pfhrp2/pfhrp3* is not known. Additional studies are needed in Africa to determine the cause of false-negative RDTs, and specifically whether, as is the case in parts of South America and in Eritrea, true deletions of *pfhrp2/3* are responsible for a substantial number of false negative diagnostic assays.

## Conclusion

In summary, false negative PfHRP2-based RDTs were uncommon in Uganda, and that false negatives were explained by non-falciparum infections, PCR amplification failures, and, only in a small subset of samples, true absence of expression of PfHRP2. Nonetheless, the prevalence of *pfhrp2/pfhrp3* deletions may be increasing in various regions [13]. As this phenomenon threatens a primary method of malaria diagnosis in Africa, continued surveillance for *pfhrp2/pfhrp3* deletions across Africa, ideally using multiple experimental methods, is a high priority. Results suggest that definitive detection of *pfhrp2/pfhrp3* deletion should incorporate assays for both DNA and protein; a multiplex format will facilitate high throughput screening.

## Abbreviations

PfHRP2: *P. falciparum* histidine rich protein 2
PfHRP3: *P. falciparum* histidine rich protein 3
*pfhrp2*: *Plasmodium falciparum histidine rich protein 2 gene*
*pfhrp3*: *Plasmodium falciparum histidine rich protein3 gene*
RDT: Rapid diagnostic test
MSP1: *Merozoite surface antigen 1*
PCR: Polymerase chain reaction
WHO: World Health Organization
DNA: Deoxyribonucleic acid
DBS: Dried blood spots
MOI: Multiplicity of infection
PBS: Phosphate buffered saline

## References

[1] WHO. World malaria report. Geneva, Global Malaria Programme, World Health Organization, 2019:232.

[2] Poti KE, Sullivan DJ, Dondorp AM, Woodrow CJ (2020). HRP2: transforming malaria diagnosis, but with caveats. Trends Parasitol..

[3] Chiodini PL, Bowers K, Jorgensen P, Barnwell JW, Grady KK, Luchavez J (2007). The heat stability of *Plasmodium* lactate dehydrogenase-based and histidine-rich protein 2-based malaria rapid diagnostic tests. Trans R Soc Trop Med Hyg.

[4] Lee N, Baker J, Andrews KT, Gatton ML, Bell D, Cheng Q (2006). Effect of sequence variation in *Plasmodium falciparum* histidine- rich protein 2 on binding of specific monoclonal antibodies: implications for rapid diagnostic tests for malaria. J Clin Microbiol.

[5] Abdallah JF, Okoth SA, Fontecha GA, Torres RE, Banegas EI, Matute ML (2015). Prevalence of pfhrp2 and pfhrp3 gene deletions in Puerto Lempira, Honduras. Malar J..

[6] Watson OJ, Slater HC, Verity R, Parr JB, Mwandagalirwa MK, Tshefu A (2017). Modelling the drivers of the spread of *Plasmodium falciparum* hrp2 gene deletions in sub-Saharan Africa. Elife..

[7] Gatton ML, Dunn J, Chaudhry A, Ciketic S, Cunningham J, Cheng Q (2017). Implications of parasites lacking *Plasmodium falciparum* histidine-rich protein 2 on malaria morbidity and control when rapid diagnostic tests are used for diagnosis. J Infect Dis.

[8] Mukkala AN, Kwan J, Lau R, Harris D, Kain D, Boggild AK (2018). An update on malaria rapid diagnostic tests. Curr Infect Dis Rep..

[9] Wurtz N, Fall B, Bui K, Pascual A, Fall M, Camara C (2013). *Pfhrp2* and *pfhrp3* polymorphisms in *Plasmodium falciparum* isolates from Dakar, Senegal: impact on rapid malaria diagnostic tests. Malar J..

[10] Houze S, Hubert V, Le Pessec G, Le Bras J, Clain J (2011). Combined deletions of pfhrp2 and pfhrp3 genes result in *Plasmodium falciparum* malaria false-negative rapid diagnostic test. J Clin Microbiol.

[11] Harvey SA, Jennings L, Chinyama M, Masaninga F, Mulholland K, Bell DR (2008). Improving community health worker use of malaria rapid diagnostic tests in Zambia: package instructions, job aid and job aid-plus-training. Malar J..

[12] Gamboa D, Ho MF, Bendezu J, Torres K, Chiodini PL, Barnwell JW, et al. A large proportion of *P. falciparum* isolates in the Amazon region of Peru lack pfhrp2 and pfhrp3: implications for malaria rapid diagnostic tests. PLoS One. 2010;5:e8091. 10.1371/journal.pone.0008091 PMC2810332 20111602

[13] Berhane A, Anderson K, Mihreteab S, Gresty K, Rogier E, Mohamed S (2018). Major threat to malaria control programs by *Plasmodium falciparum* lacking histidine-rich protein 2, Eritrea. Emerg Infect Dis..

[14] Koita OA, Doumbo OK, Ouattara A, Tall LK, Konare A, Diakite M (2012). False-negative rapid diagnostic tests for malaria and deletion of the histidine-rich repeat region of the hrp2 gene. Am J Trop Med Hyg.

[15] Kozycki CT, Umulisa N, Rulisa S, Mwikarago EI, Musabyimana JP, Habimana JP (2017). False-negative malaria rapid diagnostic tests in Rwanda: impact of *Plasmodium falciparum* isolates lacking hrp2 and declining malaria transmission. Malar J..

[16] Parr JB, Verity R, Doctor SM, Janko M, Carey-Ewend K, Turman BJ (2017). Pfhrp2-Deleted *Plasmodium falciparum* parasites in the Democratic Republic of the Congo: a national cross-sectional survey. J Infect Dis.

[17] Beshir KB, Sepulveda N, Bharmal J, Robinson A, Mwanguzi J, Busula AO (2017). *Plasmodium falciparum* parasites with histidine-rich protein 2 (pfhrp2) and pfhrp3 gene deletions in two endemic regions of Kenya. Sci Rep..

[18] Thomson R, Beshir KB, Cunningham J, Baiden F, Bharmal J, Bruxvoort KJ (2019). pfhrp2 and pfhrp3 gene deletions that affect malaria rapid diagnostic tests for *Plasmodium falciparum*: analysis of archived blood samples from 3 African countries. J Infect Dis.

[19] Bosco AB, Anderson K, Gresty K, Prosser C, Smith D, Nankabirwa JI (2020). Molecular surveillance reveals the presence of *pfhrp2* and *pfhrp3* gene deletions in *Plasmodium falciparum* parasite populations in Uganda, 2017-2019. Malar J..

[20] Katureebe A, Zinszer K, Arinaitwe E, Rek J, Kakande E, Charland K (2016). Measures of malaria burden after long-lasting insecticidal net distribution and indoor residual spraying at three sites in Uganda: a prospective observational study. PLoS Med..

[21] Nankabirwa JI, Yeka A, Arinaitwe E, Kigozi R, Drakeley C, Kamya MR (2015). Estimating malaria parasite prevalence from community surveys in Uganda: a comparison of microscopy, rapid diagnostic tests and polymerase chain reaction. Malar J..

[22] Petithory JC, Ardoin F, Ash LR (2005). Rapid and inexpensive method of diluting Giemsa stain for diagnosis of malaria and other infestations by blood parasites. J Clin Microbiol.

[23] Plowe CV, Djimde A, Bouare M, Doumbo O, Wellems TE (1995). Pyrimethamine and proguanil resistance-conferring mutations in *Plasmodium falciparum* dihydrofolate reductase: polymerase chain reaction methods for surveillance in Africa. Am J Trop Med Hyg.

[24] Snounou G, Viriyakosol S, Jarra W, Thaithong S, Brown KN (1993). Identification of the four human malaria parasite species in field samples by the polymerase chain reaction and detection of a high prevalence of mixed infections. Mol Biochem Parasitol.

[25] Cattamanchi A, Kyabayinze D, Hubbard A, Rosenthal PJ, Dorsey G (2003). Distinguishing recrudescence from reinfection in a longitudinal antimalarial drug efficacy study: comparison of results based on genotyping of msp-1, msp-2, and glurp. Am J Trop Med Hyg.

[26] Plucinski MM, Dimbu PR, Fortes F, Abdulla S, Ahmed S, Gutman J (2018). Posttreatment HRP2 clearance in patients with uncomplicated *Plasmodium falciparum* malaria. J Infect Dis.

[27] Rogier E, van den Hoogen L, Herman C, Gurrala K, Joseph V, Stresman G (2019). High-throughput malaria serosurveillance using a one-step multiplex bead assay. Malar J..

[28] Rogier E, Plucinski M, Lucchi N, Mace K, Chang M, Lemoine JF (2017). Bead-based immunoassay allows sub-picogram detection of histidine-rich protein 2 from *Plasmodium falciparum* and estimates reliability of malaria rapid diagnostic tests. PLoS ONE.

[29] Asua V, Tukwasibwe S, Conrad M, Walakira A, Nankabirwa JI, Mugenyi L (2017). *Plasmodium* species infecting children presenting with malaria in Uganda. Am J Trop Med Hyg.

